# Supplementary material for: New Insights into the Role of the Trypanosoma cruzi Aldo-Keto Reductase TcAKR
Source: Pathogens. 2023 Jan 5;12(1):85. doi: 10.3390/pathogens12010085 (PMC9860839; doi:10.3390/pathogens12010085)
Supplement: Supplementary file 1 [file Supplementary_Figure S1.pdf › Supplementary_Figure S1.pdf]

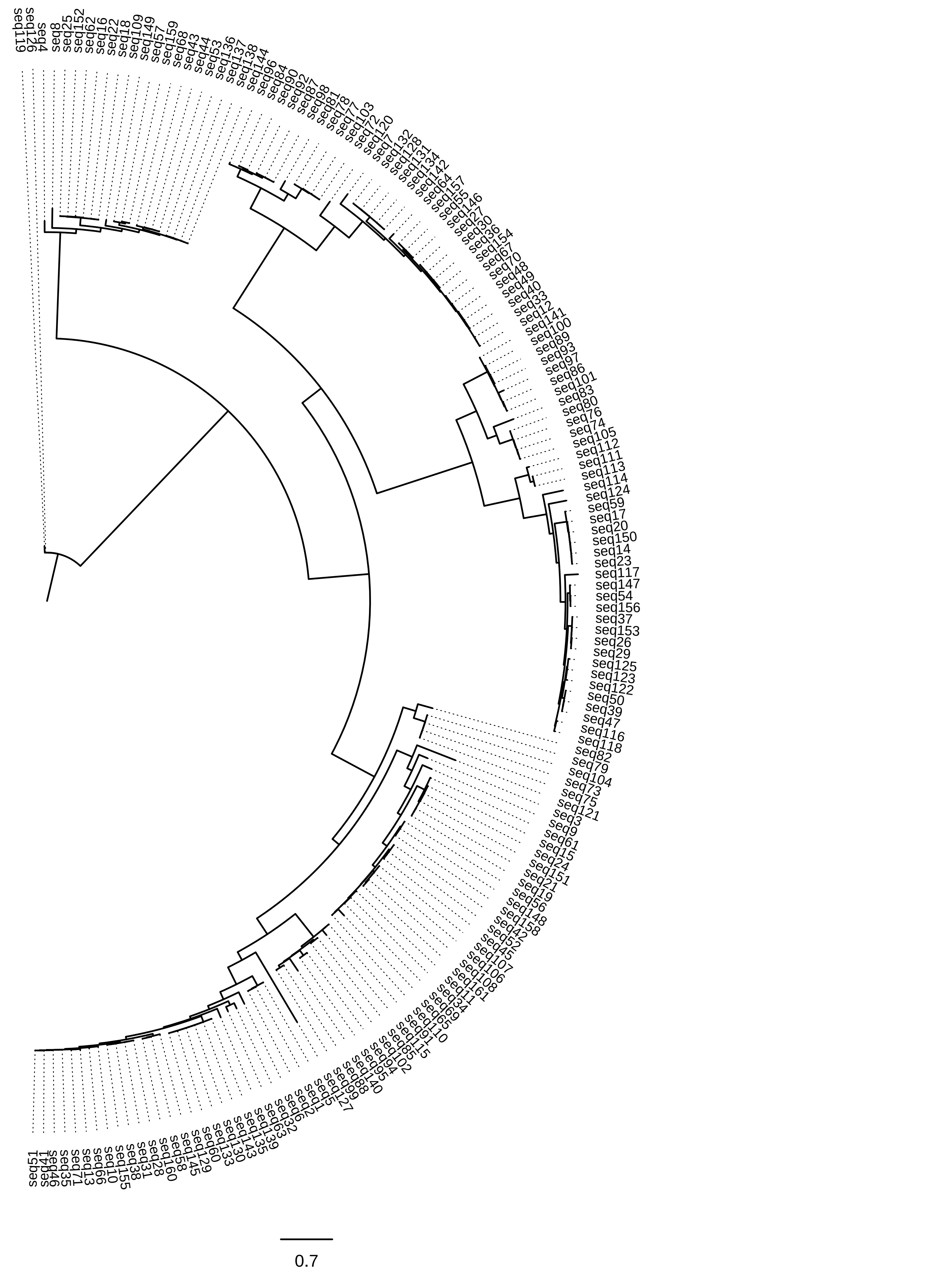

0.7
